# Occurrence and biodiversity of *Eimeria* spp. (Apicomplexa: Eimeriidae) in Madura cattle reared on Kamal Subdistrict, Madura Island, Indonesia

**DOI:** 10.14202/vetworld.2022.2084-2088

**Published:** 2022-08-27

**Authors:** Poedji Hastutiek, Nunuk Dyah Retno Lastuti, Lucia Tri Suwanti, Agus Sunarso, Dyah Ayu Kurniawati, Aditya Yudhana

**Affiliations:** 1 Department of Veterinary Science, Division of Veterinary Parasitology, Faculty of Veterinary Medicine, Universitas Airlangga, Surabaya, Indonesia; 2 Institute of Tropical Disease, Universitas Airlangga, Surabaya, Indonesia; 3 Indonesian Research Center for Veterinary Sciences, Indonesian Agency for Agricultural Research and Development, Ministry of Agriculture Republic Indonesia, Indonesia; 4 Department of Veterinary Medicine, School of Health and Life Sciences, Universitas Airlangga, Banyuwangi, Indonesia

**Keywords:** biodiversity, *Eimeria* species, infectious disease, Madura cattle, Madura Island

## Abstract

**Background and Aim:**

In Indonesia, Madura cattle are native breeds that are expected to contribute to the improvement of regional meat self-sufficiency. *Eimeria* spp. are protozoans that are commonly found in ruminants. This study aimed to identify the occurrence and diversity of *Eimeria* spp. in Madura cattle.

**Materials and Methods:**

In this study, fresh fecal samples were collected from 100 cattle in Kamal Subdistrict, Bangkalan District, Madura Island, Indonesia. Morphological detection was performed using a light microscope, and molecular identification was performed using a polymerase chain reaction. DNA amplification was conducted using various species-specific primers for *Eimeria bovis*, *Eimeria zuernii*, *Eimeria auburnensis*, *Eimeria alabamensis*, *Eimeria ellipsoidalis*, and *Eimeria cylindrica*.

**Results:**

The results obtained 21% (21/100) of *Eimeria* spp. based on morphological detection. A total of 15 positive samples with 500–25,000/mL oocysts were selected for DNA extraction and amplification, resulting in 12 positive samples. Four *Eimeria* spp. were obtained based on molecular identification: *E. bovis*, *E. zuernii*, *E. auburnensis*, and *E. cylindrica*.

**Conclusion:**

Four species of *Eimeria* namely *E. bovis*, *E. zuernii*, *E. auburnensis*, and *E. cylindrica* were identified from fecal sample of Madura cattle using PCR method in this study. Further comprehensive studies are required to investigate the pathogenicity of *Eimeria* spp. in Madura cattle. Therefore, improved and integrated management practices should be strengthened by local governments to prevent pathogenic diseases and increase national livestock productivity in Indonesia.

## Introduction

Bovine coccidiosis, caused by *Eimeria* spp., is a parasitic disease that is common in cattle. There are approximately 21 *Eimeria* species in cattle, of which *Eimeria bovis* and *Eimeria zuernii* are the most pathogenic [1]. Coccidiosis in adult animals is often asymptomatic but can be a reservoir for calves [2]. Infection in calves causes diarrhea, dehydration, dysentery, debilitation, and death in severe cases [3]. Although most of them are non-pathogenic, *Eimeria* spp. can cause intestinal tissue damage and decrease productivity in meat and milk [4]. In addition, *Eimeria* spp. infection in cattle can increase their vulnerability to other infectious diseases such as pneumonia and bacterial and viral disease [5, 6]. The economic loss due to coccidiosis in cattle was estimated at USD 400 million worldwide. In Mexico, coccidiosis reportedly affects the economics of large and small ruminants, with annual losses up to USD 23.7 million [6].

Coccidiosis cases are easily found in managed farms in dirty environments, which are contaminated by *Eimeria* oocysts. Cattle are infected with *Eimeria* spp. through ingestion of sporulated oocysts that contaminate water and feed as the main source of transmission [7]. Factors related to the prevalence of *Eimeria* spp. infection in cattle include farm management, age, and environmental temperature [8]. In Indonesia, farm management is mainly based on traditional systems managed by family units. Madura cattle are one of the main meat sources in Madura Island, Indonesia. This breed is also expected to contribute to the improvement of regional meat self-sufficiency. The manifestation of *Eimeria* spp. in the cattle might be affecting the achievement of the program.

To the best of our knowledge, there are no data regarding economic loss due to bovine coccidiosis in Indonesia because most studies only focused on poultry coccidiosis. Only a few studies have reported *Eimeria* infections, particularly in cattle in Indonesia. Ananta *et al*. [9] reported 22.4% prevalence of *Eimeria* in cattle in West Java Province, and Hamid *et al*. [10] reported 15.5% in Central Java Province. Coccidiosis in Madura cattle was also reported microscopically by Hastutiek *et al*. [11], with a prevalence of 75.07%. However, in almost studies, *Eimeria* spp. were only observed morphologically using the microscopic examination. The first report of *Eimeria* species based on molecular identification in Indonesia was done by Ekawasti *et al*. [4], who reported that the prevalence of each species was 10.4%, 2.8%, 2.1%, 1.4%, 1.1%, and 0.4%, for *E. bovis*, *Eimeria ellipsoidalis*, *Eimeria alabamensis*, *E. zuernii*, *Eimeria auburnensis*, and *Eimeria cylindrical*.

Therefore, this study aimed to identify various species of *Eimeria* spp. in Madura cattle using a molecular diagnostic approach.

## Materials and Methods

### Ethical approval

No ethical approval was required as samples were collected for diagnostic purposes only. This study not used cattle as the sample but used only fresh fecal samples that had been collected from around the enclosures. However, sample collections in the field were conducted with permission from the Animal Husbandry Department in Bangkalan District, Madura Island.

### Study period and location

The study was conducted from January to December 2020. The fecal samples of Madura cattle were collected in the Kamal Subdistrict, Bangkalan District, Madura Island. Morphological and molecular identification of *Eimeria* spp. were conducted in the Laboratory of Veterinary Parasitology, Division of Veterinary Parasitology, Department of Veterinary Science, Faculty of Veterinary Medicine, Universitas Airlangga, Surabaya, East Java Province, Indonesia. 

### Study sites and sampling methods

Kamal Subdistrict (112.72713 longitude and - 7.136996 latitude) is a part of the Bangkalan District on Madura Island. The area spans over 41.40 km^2^. Madura cattle (*Sapi Madura*) are a stable, inbred hybrid of Zebu and Banteng (*Bos* *javanicus*) [12] that originated from the island of Madura. Their body appearance is very similar to that of Bali cattle, which have the same origin as Banteng. The color is reddish-brown with non-specific white patterning on the leg and rump. Adult bulls weigh approximately 250–300 kg. A total of 100 cattle fecal samples were collected from fresh dung (<8 h) and stored in plastic bags containing potassium dichromate. No animals showed specific clinical symptoms when fecal samples were collected. Sample size calculation based on 10% of the total Madura cattle population from each village were located in north, south, east, west, and center part of Kamal Subdistrict.

### Fecal examination

The samples were analyzed using modified sugar flotation methods [13]. Sugar flotation methods were used, with a specific gravity of 1.2 (Gulaku Indonesia, Lampung, Indonesia). Approximately 2–4 g of feces was diluted with 12 mL of Aquadest. The fecal solution was filtered, and the filtrate was transferred to a 15 mL centrifuge tube. The sample was centrifuged at 3000× *g* for 10 min. The supernatant was discarded and resuspended in a sugar solution. The suspension was mixed and centrifuged at 3000× *g* for 10 min. The supernatant was collected and examined on a glass slide at 100× and 400× under alight microscope (Olympus, Guangzhou, China). *Eimeria* parasites were identified based on morphological features, such as size, shape, number of sporozoites, and other notable characteristics [14]. A qualitative microscopic examination was performed to determine the presence and absence of oocysts. A quantitative examination was performed by counting the number of oocysts per milliliter.

The purification was performed using a positive sample. *Eimeria* oocysts were purified using the sugar flotation method [15]. *Eimeria* oocysts were placed on the surface of the sugar solution using a pipette of approximately 1–2 mL. The supernatant was washed three times with distilled water. The pellet was added to 1–2 mL of PBS and stored at 4°C.

### Molecular identification

Fifteen morphologically positive samples were subjected to molecular analyses. The selection of molecular samples was based on the number of oocysts containing 250–25,000 oocysts per milliliter of fecal solution. DNA was extracted using DNAzol (Ohio, USA), according to the manufacturer’s recommended procedures. DNA was amplified using the primer pairs *Eimeria* specific (species) primers [15, 16]. Primers were specific for *E. bovis*, *E. zuernii*, *E. auburnensis*, *E. cylindrica, E. alabamensis*, and *E. ellipsoidalis*. In this study, the amplification reaction was performed in a 25 μL solution consisting of 12.5 μL of Bioline Mastermix (Bioline, Taiwan), 1 μL of each primer, 8.5 μL distilled water, and 2 μL of the DNA template. Amplification involved an initial denaturation phase at 94°C for 30 s, followed by 35 cycles at 94°C for 10 s, 52°C for 20 s, and 72°C for 20 s, and a final extension at 72°C for 2 min [15]. Then, 10 μL of polymerase chain reaction (PCR) products were electrophoresed on 1.5% agarose gel, stained with ethidium bromide, and visualized in an ultraviolet transilluminator.

## Results

**Figure-1:**
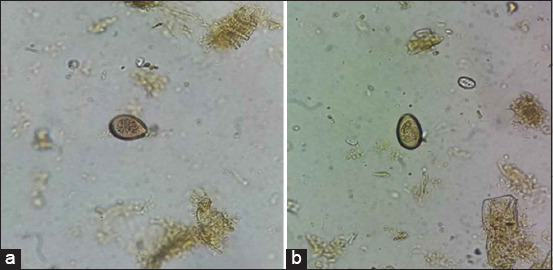
Identification of Eimeria spp. unsporulated oocyst (left) and sporulated oocyst (right) using a light microscope (400×).

**Figure-2:**
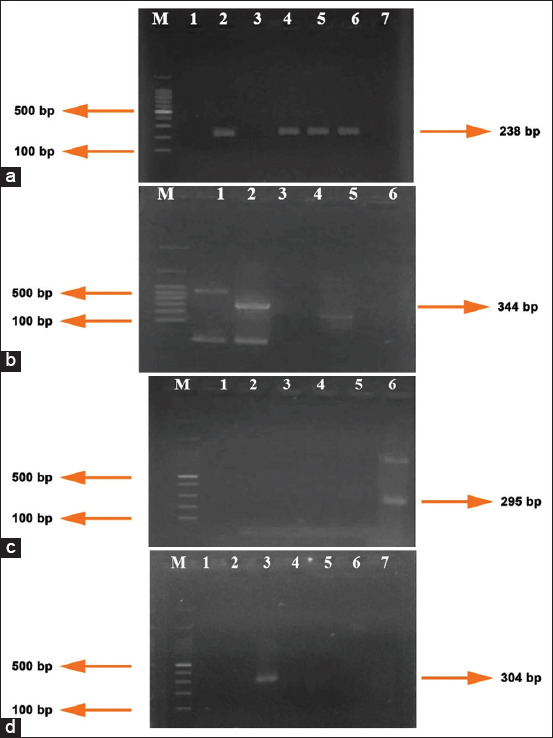
(a) Polymerase chain reaction (PCR) DNA products of *Eimeria bovis* from fecal sample of Madura cattle. M=DNA ladder; 1–7 samples. Samples 1, 3, and 7 are negative and samples 2, 4, 5, and 6 are positive. (b) PCR DNA products of *Eimeria zuernii* from fecal sample of Madura cattle. M=DNA ladder; 1–5 samples. Samples 1, 3, 4, and 5 are negative and sample 2 is positive. (c) PCR DNA products of *Eimeria auburnensis* from fecal sample of Madura cattle. M=DNA ladder; 1–6 samples. Samples 1, 2, 3, 4, and 5 are negative and sample 6 is positive. (d) PCR DNA products of *Eimeria cylindrica* from fecal sample of Madura cattle. M=DNA ladder; 1–7 samples. Samples 1, 2, 4, 5, 6, and 7 are negative and sample 3 is positive.

*Eimeria* spp. were identified in 21 of the 100 (21%) fecal samples using microscopy. The morphology of *Eimeria* spp., sporulated, and unsporulated oocysts, based on observation under a light microscope, is shown in Figure-1. Of 15 samples amplified by PCR, 12 samples were successfully amplified. The results of running PCR products showed that four *Eimeria* species were found: *E. bovis*, *E. zuernii*, *E. auburnensis*, and *E. cylindrica* (Figure-2a-d). Six samples were detected for *E. bovis*, three for *E. zuernii*, two for *Eimeria Aurburnensis*, and one for *E. cylindrica*. *E. bovis* was detected more frequently in this study. Five samples were found with a single infection and three samples with mixed infections.

## Discussion

Many studies have reported the prevalence of *Eimeria* spp. in cattle in different countries using a standard microscopy examination to detect oocysts [2, 3, 8, 10]. The prevalence was different in each country; the prevalence of *Eimeria* infection 75.5% in Colombia [3], 22.1% in South Korea [8], 47.09% in Pakistan [16], and 11.97% in India [17]. Ekawasti *et al*. [4] reported 52.3% prevalence of *Eimeria* on Java Island. Furthermore, bovine coccidiosis has also been reported in Maluku Island as the highest (94.1%) prevalence, followed by Kalimantan (83%), Sumatra (70.3%), Sulawesi (68.9%), Papua (62.3%), and Nusa Tenggara (58.5%) [18]. The variation in prevalence and type of infection can differ depending on the various infection rates and shedding intensities of individuals. These differences might be due to geographical conditions, sources of feed, and feeding behavior [19].

Based on the molecular diagnostic findings, 12 samples were shown positive. *E. bovis* was frequently found in this study, followed by *E. zuernii, Eimeria aurburnensis*, and *E. cylindrica*. In South Korea, Lee *et al*. [8] reported that *E. bovis* was identified in 79% and *E. zuernii* in 66% of samples. Ekawasti *et al*. [4] also reported that *E. bovis* (10.4%) is the most prevalent species on Java Island, Indonesia. Using PCR as a molecular approach, Lee *et al*. [8] successfully identified three species of *Eimeria*, namely, *E. bovis*, *E. zuernii*, and *E. auburnensis*. Ekawasti *et al*. [4] identified *E. bovis, E. ellipsoidalis, E. alabamensis, E. zuernii, E. auburnensis*, and *E. cylindrica*. Moreover, in this study, not all the positive samples in the microscopic examination showed positive PCR results. A possible reason for this is the limited number of oocysts in fecal samples. Those findings were supported by the statement of Carvalho *et al*. [20], Mirhashemi *et al*. [21], and Ekawasti *et al*. [4], who explained that a small number of oocysts were not sufficient for species identification using the PCR method. The presence of contaminants possibly also inhibited the PCR process during the procedures [4, 15].

Bovine coccidiosis can cause not only growth delays but also a decrease in body performance and cattle production. These clinical signs also affect the quality of adult cattle, thus resulting in high morbidity and mortality in calves, inhibiting the sustainability of livestock production [22]. Theoretically, coccidiosis is a pathogenic disease of young animals, but poor nutritional and environmental management can be potential risk factors for older animals. Adult cattle with chronic infection are frequently diagnosed with anorexia, weight loss, emaciation, bloody diarrhea, and blood-stained dung in perineum and tail part [23].

In this study, Madura cattle were infected with either single or mixed *Eimeria* species. Coccidiosis in cattle is typically caused by more than one species of *Eimeria*. The Madura cattle in this study were infected with single or mixed *Eimeria*. Bangoura *et al*. [2] reported that 48.6% of cases of diarrhea in calves were caused by a single infection, and 51.4% had mixed infections. Morgoglione *et al*. [24] also reported that 71.2% of cattle were infected with more than 1 *Eimeria* species. These previous results were different from our study, which showed that a single infection was recorded more frequently compared to mixed infections. In the sampling area, the management of cages and sanitation is also known to be improper because feces that were cleared from the cages were dumped right around the cages, which might potentially increase the risk of infection and reinfection [25, 26]. The majority of cages are also known to be traditional and still not equipped with feces and urine disposal lines.

Management patterns also affect the occurrence of *Eimeria* spp. infection, such as sanitation method, drainage system, population density, cage structure, feeding systems, and drinking sources [27]. Occurrences of infection and intensity of *Eimeria* spp. in cattle were also recorded at a lower percentage in a cage compared to pasture [28]. Therefore, cattle shed a lot of oocysts through feces in their closed cages every day during the patent period, which can increase the risk of transmission and increase the development cycle of *Eimeria* spp. The clinical signs of bovine coccidiosis frequently appear 2–3 weeks after infection in a contaminated environment condition [29].

To date, there have been rare reports of molecular investigations of *Eimeria* spp., especially in Indonesia [4, 30]. Although the number of samples in our study was limited, we revealed that the samples could be identified at the species level for *Eimeria* spp. using the molecular method. Therefore, comprehensive studies are required to further investigate the pathogenicity of *Eimeria* spp. infection in Madura cattle and improves productivity through improved and integrated livestock management practices.

## Conclusion

The occurrence of *Eimeria* spp. infection in Madura cattle in Kamal Subdistrict, Bangkalan District, Madura Island, is 12% detected by PCR using specific species primers. Moreover, this study successfully obtained four species: *E. bovis, E. zuernii, E. auburnensis*, and *E. cylindrica*. The occurrence of *Eimeria* spp. among Madura cattle should be considered because bovine coccidiosis is probably distributed in most parts of Madura Island. Based on these findings, molecular detection of coccidiosis in Madura cattle can be applied not only in one district but also in several districts, with different conditions associated with the risk factors. The biosecurity measures need to be strengthened among traditional farmers to control the transmission of *Eimeria* spp. in Madura cattle.

## Authors’ Contributions

PH, LTS, AS, and DAK: Collected fecal samples. PH, NDRL, and DAK: Analyzed the microscopic observation and molecular identification. PH, DAK, and AY: Wrote original draft and revised the manuscript. All authors have read and approved the final manuscript.
